# Frontal Functional Connectivity of Electrocorticographic Delta and Theta Rhythms during Action Execution Versus Action Observation in Humans

**DOI:** 10.3389/fnbeh.2017.00020

**Published:** 2017-02-07

**Authors:** Claudio Babiloni, Claudio Del Percio, Susanna Lopez, Giancarlo Di Gennaro, Pier P. Quarato, Luigi Pavone, Roberta Morace, Andrea Soricelli, Giuseppe Noce, Vincenzo Esposito, Vittorio Gallese, Giovanni Mirabella

**Affiliations:** ^1^Department of Physiology and Pharmacology, University of Rome “La Sapienza”Rome, Italy; ^2^IRCCS S. Raffaele PisanaRome, Italy; ^3^IRCCS SDNNaples, Italy; ^4^IRCCS NeuromedPozzilli (IS), Italy; ^5^Department of Motor Sciences and Healthiness, University of Naples ParthenopeNaples, Italy; ^6^Department of Neuroscience, University of ParmaParma, Italy

**Keywords:** subdural electrocorticography (ECoG), movement observation, movement execution, lagged linear connectivity, exact low-resolution brain electromagnetic tomography (eLORETA), frontal cortex, mirror neuron system

## Abstract

We have previously shown that in seven drug-resistant epilepsy patients, both reaching-grasping of objects and the mere observation of those actions did desynchronize subdural electrocorticographic (ECoG) alpha (8–13 Hz) and beta (14–30) rhythms as a sign of cortical activation in primary somatosensory-motor, lateral premotor and ventral prefrontal areas (Babiloni et al., 2016a). Furthermore, that desynchronization was greater during action execution than during its observation. In the present exploratory study, we reanalyzed those ECoG data to evaluate the proof-of-concept that lagged linear connectivity (LLC) between primary somatosensory-motor, lateral premotor and ventral prefrontal areas would be enhanced during the action execution compared to the mere observation due to a greater flow of visual and somatomotor information. Results showed that the delta-theta (<8 Hz) LLC between lateral premotor and ventral prefrontal areas was higher during action execution than during action observation. Furthermore, the phase of these delta-theta rhythms entrained the local event-related connectivity of alpha and beta rhythms. It was speculated the existence of a multi-oscillatory functional network between high-order frontal motor areas which should be more involved during the actual reaching-grasping of objects compared to its mere observation. Future studies in a larger population should cross-validate these preliminary results.

## Introduction

A bulk of magnetoencephalographic (MEG), electroencephalographic (EEG; Babiloni et al., 1999; Pfurtscheller and Lopes da Silva, 1999) and electrocorticographic (ECoG; Crone et al., 1998b; Miller et al., 2007) findings have shown that a desynchronization of alpha (i.e., 8–13 Hz) and beta (i.e., 15–25 Hz) oscillations, which constitute the so-called mu rhythm, occurs not only when passive or active movements are performed, as a reflection of underlying somatomotor information processing, but also during the observation of actions performed by other people, as a reflection of underlying visual information processing (Pineda, 2005). Specifically, Gastaut and Bert (1954) reported that central EEG mu rhythm desynchronizes during the observation of films depicting biological motion (e.g., a bike race, boxing) as a function of the identification with the actor on screen. This finding was corroborated by further MEG and EEG evidence (for a review see Pineda, 2005). For instance, it has been reported that MEG and EEG beta synchronization following median nerve stimulation decreased in magnitude in a dipolar model of the activity in primary motor cortex during both movement execution and observation (Hari et al., 1998; Rossi et al., 2002; Muthukumaraswamy and Johnson, 2004a). Furthermore, EEG alpha and beta rhythms desynchronized over widespread frontal, central and parietal areas during the observation of movements performed by other people (Cochin et al., 1998, 1999; Babiloni et al., 2002; Martineau and Cochin, 2003; Neuper et al., 2009; Perry and Bentin, 2009).

Some other studies have shown a high degree of specialization of this mechanism, by demonstrating a strict functional relationship between the mu rhythm and movement parameters. For instance, it was found that the observation of a precision grip induced a stronger frontal and parietal alpha desynchronization when compared to observing a flat-hand extension or a precision grip gesture with no object to grasp (Muthukumaraswamy and Johnson, 2004b; Muthukumaraswamy et al., 2004). This finding can be considered as the “negative” counterpart (i.e., suppression of a background EEG oscillatory activity) of the binding processes that couple human non-primary frontoparietal “mirror” neurons and primary somatomotor neurons during movement observation (Pineda, 2005). In the same vein, frontal beta rhythms were associated with kinematic features of reaching and grasping objects executed by others, as an evidence of its strict relationship with visuomotor transformation processes (Avanzini et al., 2012). Beta rhythms were also related to kinematic features of observed movements in monkeys, as revealed by parietal local field potentials at beta frequencies (Kuang et al., 2016). During action observation, the desynchronization of the mu rhythm is not associated with a synchronization at 40 Hz (Pfurtscheller and Lopes da Silva, 1999; Perry et al., 2010; Wriessnegger et al., 2013), which can be considered as the “positive” counterpart of the above mentioned binding processes between mirror neurons.

However, non-invasive scalp EEG and MEG techniques have an insufficient spatial resolution to disentangling the effects of a movement observation on mu rhythms generated by contiguous primary somatosensory, primary motor and premotor areas. Therefore, this issue has been addressed by subdural ECoG recordings performed during the pre-surgical monitoring in drug-resistant epilepsy patients, which are characterized by high spatial and temporal resolution. One study, done just in one patient, has shown an alpha desynchronization in primary motor and Broca’s area during both execution and observation of aimless movements (Tremblay et al., 2004). No report concerning gamma synchronization was reported (Tremblay et al., 2004). To overcome these limitations, we recorded subdural ECoG activity from seven drug-resistant epilepsy patients, to assess whether both movement observation and execution induce parallel modulations of alpha, beta and gamma rhythms in task-relevant sensorimotor cortical regions (Babiloni et al., 2016a). In the two conditions (i.e., movement execution and observation), common objects implying power grips (e.g., cup, phone, glasses) were reached and grasped. Both arm-hand action execution and observation induced a desynchronization of alpha (8–12 Hz) and beta (16–24 Hz) rhythms in primary somatosensory (Brodmann area, BA, 1–2), primary motor (BA4), lateral premotor (BA6) and the inferior frontal gyrus (BA44 and BA45). This desynchronization was generally higher in amplitude during the execution than the observation of the actions, likely as a consequence of higher cortical activation during the former than during the latter condition. Action execution also induced a synchronization of gamma (36–44 Hz) rhythms in BA4 and BA6, as an additional sign of enhanced cortical activation. These results agree with previous evidence showing frontal alpha and beta desynchronization during the observation of movements performed by other people (Perry et al., 2010; Wriessnegger et al., 2013). As a novelty, Babiloni et al. (2016a) provided fine spatial details on the multiple oscillatory neurophysiological mechanisms underlying the binding of visual, premotor and prefrontal areas during the observation of an act as well as during the execution of the same act.

It may be argued that the above EEG and MEG results do not capture a main feature of human higher cognitive-motor functions, namely the linear functional or effective connectivity within long-range brain networks (Varela et al., 2001; Le Van Quyen et al., 2003; Börner et al., 2007). It can be speculated that most cortical processes related to action OBSERVATION and EXECUTION would be highly distributed and synchronized across several cortical and sub-cortical sensorimotor regions. Therefore, an ideal methodological approach would be the extraction of some indexes of functional brain connectivity from ECoG rhythms at electrode pairs. In this framework, linear indexes such as spectral coherence would provide information about the phase relationship between the EEG or ECoG rhythms recorded at electrode pairs (Rappelsberger and Petsche, 1988; Gerloff et al., 1998; Gevins et al., 1998). Other relevant linear indexes are lagged linear connectivity (LLC), Granger causality and related measures, and phase-amplitude cross-frequency coupling (CFC). LLC has the advantage to removing the zero-lag component of EEG/ECoG spectral coherence, which is likely due to the instantaneous physical propagation of the neural currents into the head as a volume conductor (Pascual-Marqui, 2007a,b,c). Granger causality and directed transfer function (DTF) would estimate the directionality of that EEG/ECoG functional coupling, suggesting the underlying effective “causal” connectivity (Babiloni et al., 2016a,b,c). Phase-amplitude CFC at a given electrode pair models the dependence between the phase of a low-frequency EEG/ECoG coupling and the amplitude of that coupling at higher EEG/ECoG frequencies (Canolty et al., 2006; Demiralp et al., 2007; Osipova et al., 2008; Cohen et al., 2009).

Therefore, the aim of the current exploratory study was to produce a proof-of-concept that event-related LLC (ErLLC) of ECoG rhythms would increase during the action execution with respect to the mere observation of the same act due to a higher flow of visual, executive motor commands and feedback somatosensory reafferent information. To this aim, the analysis of the ECoG rhythms was extended to delta/theta rhythms (<8 Hz). Previous ECoG studies in rodents have shown that these low-frequency rhythms were involved in the functional brain connectivity of distributed frontal, parietal and sub-cortical cognitive-motor networks (Jones and Wilson, 2005; Sirota et al., 2008), possibly to allow the integration of motor commands with incoming multimodal feedback sensory information (Caplan et al., 2003; Ekstrom et al., 2005). Similarly, studies on humans, unveiled that a desynchronization of theta (4–8 Hz) rhythms occurred both during the execution of reaching and grasping an object with the hand and during the observation of similar actions (i.e., the observation of video clips showing reaching and grasping an object with the hand; Frenkel-Toledo et al., 2013). Finally, it has also been shown that children exhibit higher theta desynchronization during the observation of a human movement compared to the observation of a non-biological movement (Cochin et al., 2001; Martineau et al., 2010).

## Materials and Methods

Details on the subjects, the cognitive-motor task, and ECoG recordings were reported in Babiloni et al. (2016a). In the following sections, we provide a short description of those methodological procedures.

### Subjects

Seven right-handed, adult volunteers (3 females, 4 males; age of 47.5 years ±5.9 standard error, SE) with intractable epilepsy were included in the experimental group. All ethical procedures were followed. Epileptogenic brain regions were localized following a validated procedure (Quarato et al., 2005). Apart epilepsy, no other neurological abnormality was observed.

### ECoG Acquisition and Preliminary Data Analysis

All patients, but two had (subdural) ECoG electrode grids with 64 recording contacts while the remaining two patients had grids with 48 recording contacts. Kind and localization of grids were based exclusively on clinical considerations. Specifically, electrodes were placed in ventral prefrontal (BA44 and BA45, BA44/45), lateral premotor (BA6) and primary somatosensory-motor (BA1, BA2 and BA4, BA1/2/4) areas. The electrodes of interest for this study did not show epileptogenic foci and covered the neural networks coding for the execution of visual-guided movements and understanding of similar movements performed by others, as shown by previous fMRI studies (Buccino et al., 2004; Calvo-Merino et al., 2005, 2006; Cross et al., 2006). Subdural ECoG electrodes were localized as described elsewhere (Miller et al., 2007; Mattia et al., 2012).

ECoG data acquisition was performed with a 400 Hz sampling and automatic anti-aliasing filter. All exploring electrodes were referenced to a reference electrode located at the extra-cephalic mastoid bones. Furthermore, electromyogram (EMG) of the flexor digitorum superficialis of the dominant hand, the electrocardiogram (EKG), and electrooculogram (EOG) were used for control purposes.

During the recordings of ECoG, a patient was seated in his/her bed and two experimenters were near him or her. One did seat in front of the patient, while the other was placed on the right of the patient. In OBSERVATION or EXECUTION condition, each trial started when experimenter I positioned a common object that differed in size, shape and function (cup, glass, or phone) on the plate of a mechanical device, which was placed on a small table between the patient and the experimenter II. After placing the object, the experimenter I gave a verbal instruction, which was either “Observe” or “Execute”. In the OBSERVATION condition, the experimenter II reached, grasped and lifted the object following its more common affordances, while the patient stayed still. In the EXECUTION condition, the same action was performed by the patient, while the experimenter II stayed still. In both conditions, the mechanical device detected the first lifting of the object on the plate sensor and released a TTL pulse (reduced in amplitude by a voltage divider and decoupled through an optoisolator chip) to the ECoG data acquisition system signaling the zero-time of each trial. Afterward, the object was put back to the table by the agent (i.e., patient or experimenter II) and took away by the experimenter I, who changed the objects trial-by-trial in a pseudorandom order. It can be estimated that the time gap between the verbal command given by the experimenter I and the so-called zerotime was of about 2 s. The inter-trial interval was never less than 15–20 s. The OBSERVATION and the EXECUTION condition included 60 trials each.

ECoG, EOG and EMG continuous records were divided in single epochs lasting from −5 to +5 s where zero time coincided practically with the contact between the subject’s hand and the object. The ECoG epochs with artifacts (ocular, muscular, others) were identified and rejected by the procedure validated in Moretti et al. (2003). Two experts validated that procedure.

### Estimation of Functional Connectivity of ECoG Rhythms: eLORETA LLC

The exact low-resolution brain electromagnetic tomography (eLORETA) freeware (Pascual-Marqui, 2007a) is an evolution of LORETA and standardized LORETA package (Pascual-Marqui et al., 1994, 2002) and was used to estimate the functional linear connectivity of the ECoG rhythms in the present study. Specifically, we used the LLC function (Pascual-Marqui et al., 2011), which removes the zero-lag coherence of the functional connectivity typically due to the physical propagation of the neural currents in the head as a volume conductor (Pascual-Marqui, 2007b). Furthermore, the LLC minimizes the “common feeding” phenomenon (Pascual-Marqui, 2007c), namely the instantaneous coherence between two cortical sources merely due to the effect of a third source.

In the principal analysis, the LLC of the ECoG rhythms was evaluated only between pairs of large cortical zones having direct anatomical connections. For this purpose, we preliminarily grouped cortical areas based on the classical distinction as “primary”, “secondary” and “tertiary” (Luria, 1973). Specifically, the primary somatosensory (BA1/2) and motor (BA4) areas were collapsed into a primary somatosensory-motor ROI (BA1/2/4). Furthermore, the inferior prefrontal areas (BA44 and BA45) were collapsed into a ventral prefrontal ROI (BA44/45). Finally, the lateral premotor area was considered as a unique ROI. Afterward, the LLC was computed between the BA1/2/4 and BA6 pair (six patients) and between the BA6 and BA44/45 pair (six patients). Both of them have direct anatomical connections according to previous investigations (Catani et al., 2012a,b; Thiebaut de Schotten et al., 2014). On the one hand, this choice was due to the need of reducing the number of statistical comparisons in the present small group of epilepsy patients. On the other hand, this approach, in principle, would unveil the modulation of a functional network from primary somatosensory-motor to lateral premotor areas, as well as from lateral premotor to prefrontal areas.

### Computation of Event-Related LLC (ErLLC) at a Given Electrode Pair and Frequency

The ECoG LLC for a given electrode pair and frequency was calculated during two different periods: (i) the pre-event period; and (ii) the event period. The event period was defined as the period of movement execution or observation (from 1 s before to 1 s after the zero-time, i.e., the lifting of the object), while the pre-event period was defined as the time interval between −4 s and −3 s before the zero-time.

To take into account the inter-subject variability of the ECoG LLC, we used the index named “event-related lagged linear connectivity” (ErLLC). At a given electrode pair, ErLLC was defined as the arithmetical difference between the ECoG LLC value at the event period and the ECoG LLC value at the pre-event period. It should be remarked that the magnitude of ECoG ErLLC is smaller than the absolute ECoG LLC, as it results from the difference between coherence values.

The ErLLC assumes positive values when the ECoG LLC is higher during the event than the pre-event period (i.e., enhanced ECoG LLC as a reflection of the enhanced functional connectivity of ECoG rhythms). On the contrary, the ErLLC is negative when the ECoG LLC is lower during the event than the pre-event period (i.e., reduced ECoG LLC as a reflection of the reduced functional connectivity of ECoG rhythms).

### Computation of ErLLC at Frequency Bands of Interest and Representative Electrode Pairs

We performed the ECoG ErLLC analysis at the delta-theta, alpha, beta and gamma frequency bands. A finer analysis of alpha, beta and gamma sub-bands was not performed due to the need of reducing the number of statistical comparisons given the small number of patients.

A fixed delta-theta band ranged between 2 Hz and 6 Hz to avoid possible inflating effects of ECoG activity pass-banded to 0 Hz.

For the alpha band, the alpha “reactive” frequency (ARF) was identified subject-by-subject for any BA-ROI pair (i.e., BA1/2/4 and BA6 pair; BA6 and BA44/45 pair). The ARF was defined as the frequency showing the maximum ErLLC (i.e., the maximum increase of LLC during the event period when compared to the pre-event period) within the band 7–13 Hz. All electrode pairs of a given BA-ROI pair were considered one-by-one. Based on the ARF, the alpha band ranged between ARF−2 Hz and ARF+2 Hz.

For the beta band, the beta “reactive” frequency (BRF) was identified subject-by-subject for any BA-ROI pair. The BRF was the frequency showing the maximum ErLLC within the 16–24 Hz band. All electrode pairs of a given BA-ROI pair were considered one-by-one. Based on the BRF, the beta band ranged between BRF-2 Hz and BRF+2 Hz.

A fixed gamma band varied between 36 Hz and 100 Hz (Crone et al., 1998a; Szurhaj et al., 2006; Miller et al., 2007, 2010). Noteworthy, the gamma frequency band of interest had the highest frequency limits well lower than the Nyquist frequency of 200 Hz, which is half of the sampling rate used for the digitalization of the EEG data.

Table 1 reports the mean values (± standard error mean, SE) of the ARF and BRF for each BA-ROI pair and experimental condition (i.e., EXECUTION, OBSERVATION). For each BA-ROI pair, a *t*-test evaluated whether the ARF and BRF varied between the EXECUTION and the OBSERVATION condition (*p* < 0.05). No statistically significant difference was found (*p* > 0.05).

**Table 1:** **Mean values ± standard error mean (SE) of the alpha “reactive” frequency (ARF) and the beta “reactive” frequency (BRF) peaks at two pairs of Brodmann area (BA) or cortical region of interest (ROI; i.e., BA1/2/4 and BA6 pair; BA6 and BA44/45 pair) for the EXECUTION and the OBSERVATION condition**.

	EXECUTION		OBSERVATION	
**BA- ROI pair**	**ARF (Hz)**	**BRF (Hz)**	**ARF (Hz)**	**BRF (Hz)**
BA1/2/4 and BA6	8.8 ± 0.4	19.8 ± 0.9	9.9 ± 1.0	19.9 ± 0.7
BA6 and BA44/45	10.8 ± 0.7	20.5 ± 0.5	9.2 ± 0.9	19.8 ± 0.7

*See the “Materials and Methods” Section for an explanation of the meaning of the ARF and BRF and the description of the two experimental conditions*.

For each BA-ROI pair, we computed an ErLLC value for each frequency band (i.e., delta-theta, alpha, beta and gamma), electrode pair, and experimental condition (i.e., EXECUTION or OBSERVATION). To take into account the inter-subject variability of the ECoG LLC, we identified a “*reactive* electrode pair” for a given BA-ROI pair, condition and frequency band. The reactive electrode pair was defined as the one showing the maximum ErLLC value in the BA-ROI pair for a given condition and frequency band. For example, for the BA1/2/4 and BA6 pair and a given condition, we identified (i) the delta-theta reactive electrode pair as the one showing the highest delta-theta ErLLC value, (ii) the alpha reactive electrode pair as the one showing the highest alpha ErLLC value, (iii) the beta reactive electrode pair as the one showing the highest beta ErLLC value; and (iv) the gamma reactive electrode pair as the one showing the highest gamma ErLLC value. This procedure was followed for both experimental conditions (i.e., EXECUTION and OBSERVATION) and BA-ROI pairs (i.e., BA1/2/4 and BA6; BA6 and BA44/45).

### Statistics

In this study, the number of epilepsy patients (*N* = 7) was too low when compared to the factors and levels of an ideal ANOVA design using two conditions × connectivity in two pairs of regions × four ECoG frequency bands of interest (yielding to 16 contrasts). Therefore, the statistical design included only eight *t*-tests (exploratory alpha value set at *p* = 0.05) to evaluate whether the ErLLC of ECoG rhythms would differ between the EXECUTION and the OBSERVATION condition for two pairs of regions (i.e., BA1/2/4 and BA6 pair; BA6 and BA44/45 pair) and four frequency bands (i.e., delta-theta, alpha, beta and gamma). Due to problems occurred during ECoG recording, one subject were excluded from the sample, thus limiting the degree of freedom used for all the following statistical analysis to five. These statistical analyses were performed using the software named Statistica^®^ (10.0 packages, StatSoft^®^ Inc., Tulsa, OK, USA).

Five additional analyses were conducted to control and corroborate the statistical effects of the principal analysis.

First, we assessed whether the statistical effects could be due to some outliers in the delta-theta ErLLC in one or both conditions (Grubbs’ test; alpha value *p* = 0.05).

Second, we verified whether the statistical effects could be merely due to the local event-related ECoG power density (using the pre-event period as a reference) reported in a mentioned reference study (Babiloni et al., 2016a). To address this last issue, a step-wise procedure was used. First, two *t*-tests (*p* < 0.05) evaluated possible statistically significant differences between the two conditions in the event-related ECoG power density computed at the BA-ROI most reactive electrodes the event-related ECoG power density was calculated with the formula of event-related desynchronization/synchronization (ERD/ERS- used in Babiloni et al., 2016a). Second, a correlation analysis (Pearson test, *p* < 0.05) was performed between the ErLLC at the BA-ROI pairs and the ERD/ERS computed at the same representative electrodes.

Third, we used a *t*-test to evaluate whether the ECoG LLC in the BA-ROI pairs (the most reactive electrodes) would be greater during the EXECUTION than during the pre-event period (alpha value *p* = 0.05 one-tailed).

Fourth, we evaluated whether the ErLLC effects in the BA-ROI pairs (the most reactive electrodes) would be related to a phase-amplitude coupling of the ECoG rhythms. Specifically, the hypothesis was that the phase of the delta-theta ErLLC in a cortical region would modulate the amplitude of alpha, beta and gamma rhythms in the other cortical region. To test this hypothesis, we used the eLORETA freeware to calculate the event-related phase-amplitude cross-frequency lagged connectivity (ErCFLC) in the BA-ROI pairs in both experimental conditions. The ErCFLC values were computed for two directions and three coupled frequency bands. The direction from one region to another indicated the modulation induced by the phase of the low-frequency ErCFLC in the first region on the amplitude of the ErCFLC in the second region and vice versa. The statistical significance of the effects was probed with *t*-tests (*p* < 0.05; two direction × three coupled frequency bands of interest). The hypothesis was that the ErCFLC would be greater in the EXECUTION compared to the OBSERVATION condition.

Finally, we tested whether the ErLLC would be higher at the BA1/2/4 and BA44/45 pair (two ROIs not having known direct anatomical connections) during the EXECUTION than the OBSERVATION condition. This pair was represented by the most reactive electrode. To this aim, we performed four *t*-tests (alpha value *p* = 0.05, one-tailed), one for each frequency band of interest.

## Results

**Figure 1:**
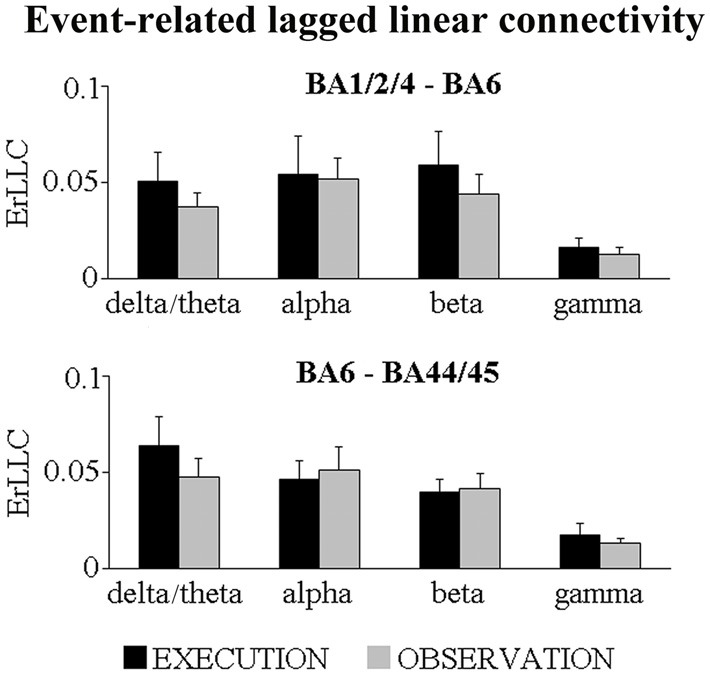
**Mean (± standard error mean, SE) values of the event-related lagged linear connectivity (ErLLC) of subdural electrocorticographic (ECoG) rhythms for two pairs of cortical region of interest (ROI; i.e., BA1/2/4 and BA6 pair; BA6 and BA44/45 pair).** The ErLLC values refer to four ECoG frequency bands of interest (i.e., delta-theta, alpha, beta, gamma) and two experimental conditions (i.e., EXECUTION, OBSERVATION). Noteworthy, the only significant *t*-test effect was that the delta-theta ErLLC in the BA6 and BA44/45 pair was greater during the EXECUTION than the OBSERVATION condition (*t* = 2.26; *p* = 0.04; one-tailed).

Figure 1 shows that the BA-ROI pairs (i.e., BA1/2/4 and BA6; BA6 and BA44/45) and conditions (i.e., EXECUTION and the OBSERVATION) had positive ErLLC values at the delta-theta, alpha, beta and gamma bands, indicating that functional connectivity of the ECoG rhythms was enhanced during the event compared to the pre-event period. The results of the *t*-tests comparing the ErLLC between the two conditions showed a statistically significant difference only for the BA6-BA44/45 pair at the delta-theta band. Specifically, the ErLLC was greater during the EXECUTION compared to the OBSERVATION (*t* = 2.26; *p* = 0.04; one-tailed).

The results of control analyses are reported in the following paragraphs.

**Figure 2:**
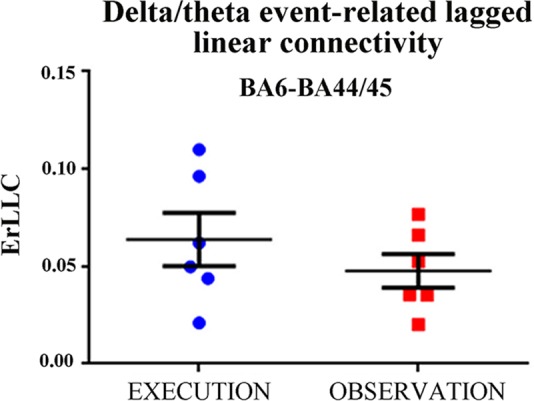
**Individual values of the delta-theta ErLLC at the BA6-BA44/45 pair for the EXECUTION and the OBSERVATION condition across all patients.** Noteworthy, the Grubbs’ test showed no outlier (*p* < 0.0001; see text for more details). Lines represent the mean (±SE) values.

First, we evaluated whether the above statistical effect in the BA6- and BA44/45 pair would be due to some outliers in the delta-theta ErLLC Figure 2 plots the individual delta-theta ErLLC values for the BA6-BA44/45 pair and both conditions. No outliers were found even when using a more stringent statistical thresholds (i.e., alpha value *p* = 0.001).

**Figure 3:**
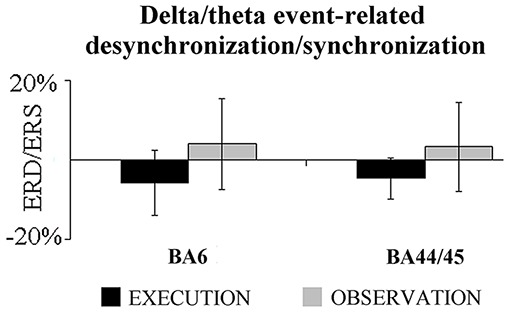
**Mean (±SE) values of the delta-theta event-related desynchronization/synchronization (ERD/ERS) at the BA6 and BA44/45 most reactive electrodes for the EXECUTION and the OBSERVATION condition.** Noteworthy, the *t*-test showed no statistically significant difference in this ERD/ERS between the two conditions (*p* > 0.15).

Second, we evaluated whether there was a statistically significant difference between the two conditions in the delta/theta event-related ECoG power density computed at the BA6 and BA44/45 at the most reactive electrodes. As shown in Figure 3, there was no difference between the two conditions (*t*-test, *p* > 0.15). The correlation analysis performed between the delta/theta ErLLC at the BA6-BA44/45 pair and the delta/theta ERD/ERS computed at the same representative electrodes. Again, no statistically significant correlation was found (*p* > 0.2). These findings suggest that the regional ECoG power density did not affect the ErLLC values.

**Figure 4:**
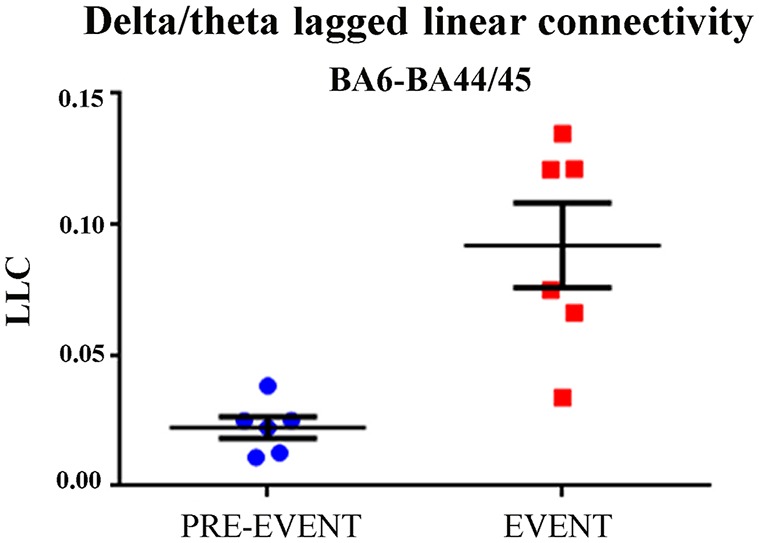
**Individual and mean (±SE) values of the delta-theta lagged linear connectivity (LLC) at the BA6 and BA44/45 pair for the pre-event and the event period in the EXECUTION condition.** Noteworthy, the *t*-test showed that the LLC was significantly higher during the EXECUTION than the pre-event period (*t* = 4.75; *p* = 0.002; one-tailed). Lines represent the mean (±SE) values.

Third, we evaluated whether the delta-theta ECoG LLC in the BA6-BA44/45 pair was greater during the EXECUTION than during the pre-event period. As shown in Figure 4, the delta-theta ECoG LLC in the BA6-BA44/45 pair was significantly greater during the EXECUTION than the pre-event period (*t* = 4.75; *p* = 0.002, one tailed).

**Figure 5:**
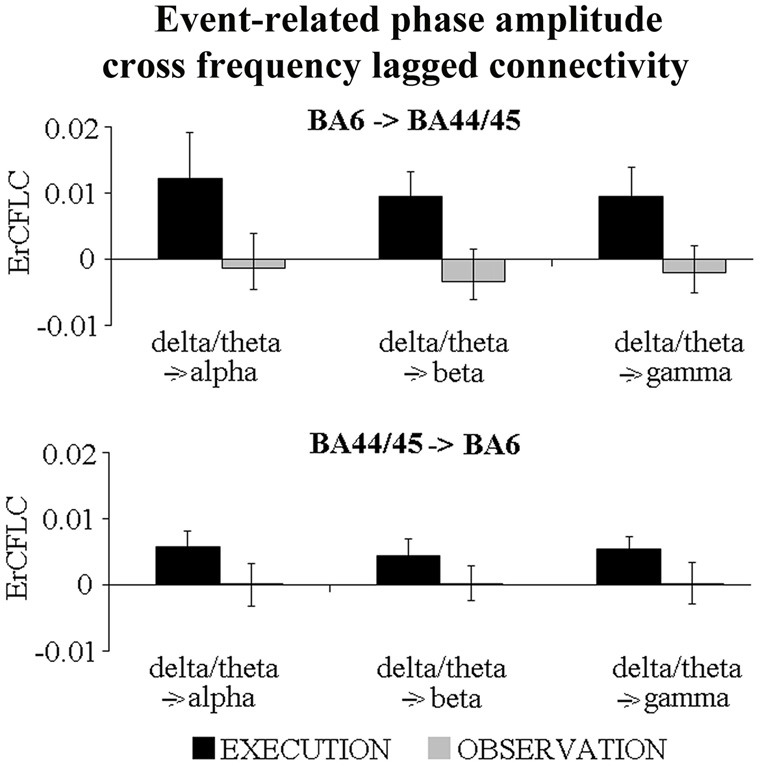
**Mean values (±SE) of the event-related phase-amplitude cross-frequency lagged connectivity (ErCFLC) values in the BA6-BA44/45 pair in the EXECUTION and the OBSERVATION condition.** These values were computed for two directions and three coupled frequency bands. ErCFLC values were always positive in the EXECUTION condition, and near to zero in the OBSERVATION condition (see text for more details). Noteworthy, the *t*-tests showed that ErCFLC values were greater during the EXECUTION compared to the OBSERVATION only in the direction from BA6 to BA44/45 (delta/theta → alpha (*t* = 3.89; *p* = 0.005; one-tailed), delta/theta → beta (*t* = 3.00; *p* = 0.014; one-tailed) and delta/theta → gamma (*t* = 3.45; *p* = 0.009; one-tailed)).

Fourth, we evaluated whether the ErLLC values could be related to a phase-amplitude coupling. Specifically, we tested whether the delta/theta phase in one cortical region may modulate the alpha (delta/theta → alpha), beta (delta/theta → beta) and gamma (delta/theta → gamma) amplitudes in the other region. Figure 5 illustrates the mean values of this phase-amplitude coupling in the EXECUTION and in the OBSERVATION condition. The execution condition was always characterized by positive ErCFLC values reflecting an enhanced phase-amplitude cross-frequency connectivity of the ECoG rhythms during the event than during the pre-event period. In contrast, the ErCFLC values in the OBSERVATION condition were always near to zero. Therefore, for both directions and for all coupled frequency bands, the ErCFLC values were higher in the EXECUTION than in the OBSERVATION condition. The statistical significance of these phenomena was probed with six *t*-tests (two directions × three coupled frequency bands; *p* < 0.05). We found that the ErCFLC values were greater during the EXECUTION compared to the OBSERVATION only in the direction from BA6 to BA44/45 (delta/theta → alpha (*t* = 3.89; *p* = 0.005; one-tailed), delta/theta → beta (*t* = 3.00; *p* = 0.014; one-tailed) and delta/theta → gamma (*t* = 3.45; *p* = 0.009; one-tailed)). These findings supported the results obtained with the ErLLC, indicating a directional coupling and unveiling a modulation induced by the phase of delta-theta ErCFLC in the BA6 on the amplitude of the ErCFLC in the BA44/45.

**Figure 6:**
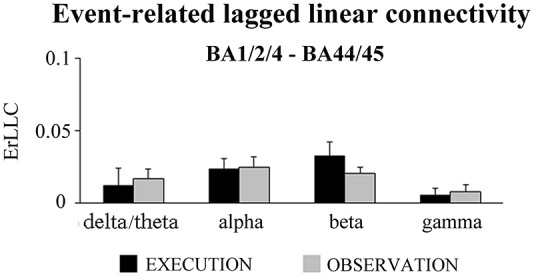
**Mean (±SE) values of the ErLLC in the EXECUTION and the OBSERVATION condition for the BA1/2/4-BA44/45 pair and the four frequency bands of interest (i.e., delta-theta, alpha, beta and gamma).** The ErLLC was computed at the most reactive electrode pair among the BA-ROI pairs. Noteworthy, the only statistically significant *t*-test effect was that the beta ErLLC was greater during the EXECUTION than the OBSERVATION condition (*t* = 2.00; *p* = 0.03; one-tailed).

Finally, we tested whether the ErLLC was higher in the EXECUTION than the OBSERVATION condition for the BA1/2/4-BA44/45 pair, two ROIs which do not have direct anatomical connections. As reported in Figure 6, ErLLC values were positive in both conditions at all frequency bands. However a significant difference was found only in the beta band (*p* < 0.05). Specifically, the beta ErLLC was greater during the EXECUTION with respect to the OBSERVATION condition (*t* = 2.00; *p* = 0.03; one-tailed).

## Discussion

In the present exploratory study, we re-analyzed the ECoG data recorded in a small group of drug-resistant epilepsy patients (Babiloni et al., 2016a). As a novelty, here we tested the proof-of-concept that the ErLLC of ECoG rhythms would be enhanced in primary somatosensory-motor, lateral premotor and ventral prefrontal areas during the actual reaching and grasping of objects in the first person compared to the mere observation of those actions performed by another person. Results are discussed in the following sections. Of note, the interpretation of those results should take into account the typical methodological pros and cons of ECoG recordings done in drug-resistant epilepsy patients. The main aim of ECoG recordings is the localization of epileptogenic brain regions for the evaluation of possible surgical removal of epileptogenic tissues. Secondary to the clinical aim, ECoG recordings are also valuable in the field of research in order to investigate neurophysiological mechanisms underpinning cognitive and sensorimotor functions in brain areas outside the epileptogenic region, typically located around the epileptogenic foci monitored. This approach provided enlightening evidence on neurophysiological mechanisms underlying the execution of visuospatial, episodic memory, language, emotional and cognitive tasks (Mukamel and Fried, 2012; Ritaccio et al., 2014; Daitch et al., 2016; Nourski et al., 2016). Compared to non-invasive EEG recordings from the human scalp, ECoG recordings have a higher spatial resolution, better gamma frequency resolution and enhanced signal-to-noise ratio (Mukamel and Fried, 2012). Furthermore, ECoG recordings are much less affected from eye movements and head muscle artifacts (Kovach et al., 2011). However, an important limitation of ECoG techniques is that epilepsy might induce some adaptive changes in the neurophysiological oscillatory mechanisms underpinning cognitive-motor functions even in the brain zones which are not producing epileptic activity. Therefore, the full understanding of these mechanisms requires the integration and comparison with findings coming from different approaches such as high-resolution scalp EEG, coregistration of scalp EEG and functional magnetic resonance imaging, MEG.

### Delta and Theta Rhythms Underpin Functional Connectivity Between Lateral Premotor and Ventral Prefrontal Areas during Action Execution Compared to Its Mere Observation

In the present study, the delta-theta (<6 Hz) ErLLC indicated that the event-related functional connectivity between the lateral premotor (BA6) and ventral prefrontal (BA44/45) areas was greater during the execution of transitive actions than during the mere observation of those actions performed by another agent. At this early stage of the research, we cannot provide a conclusive explanation for this finding, but we can provide a tentative explanation based on the available literature.

In previous works, low-frequency ECoG oscillations (3–8 Hz) recorded from the mammalian brain has been conceived as a neurophysiological mechanism for the synchronization and integration of neural activity within distributed functional networks spanning thalamocortical-cortical systems and ascending brainstem and diencephalon-septal systems, both converging on entorhinal cortex and hippocampus for the formation of memory traces (Kirk and Mackay, 2003; Vertes et al., 2004).

Previous ECoG studies in drug-resistant epilepsy patients have confirmed the importance of this mechanism based on theta oscillatory activity for short- and long-term memorization. Those ECoG studies showed that theta rhythms increased in amplitude in occipital, parietal and temporal cortical areas during short-term memory trials (Raghavachari et al., 2001). This increase in amplitude was coherent with that recorded at near cortical sites, suggesting the existence of local generating mechanisms of cortical theta rhythms (Raghavachari et al., 2006). Concerning the long-term memory, previous ECoG studies unveiled that the encoding of events to be recalled after hours was associated with an increase of frontal and temporal theta and gamma power (Sederberg et al., 2003). Furthermore, there was an increase of theta coherence between the hippocampus and rhinal-paleocortex (Fell et al., 2003), and among hippocampus, amygdala and occipital-temporal neocortex (Babiloni et al., 2009). In addition, an increase of low-frequency ECoG rhythms was observed across several cortical areas including temporal cortex during a spatial learning task implying the navigation into computerized mazes (Kahana et al., 1999). Finally, a widespread increase of cortical theta rhythms during the encoding of items subsequently recalled was also reported from scalp EEG recordings in healthy adults (Klimesch et al., 1996; Weiss et al., 2000; Sauseng et al., 2010).

Although this bulk of literature focused on the relationship between the brain theta rhythms and memory, the modulation of those rhythms in distributed functional networks is unlikely to represent a peculiar neural underpinning of episodic memory. Rather, neural synchronization at low-frequencies spanning delta and theta (<8 Hz) might be viewed as a general neurophysiological mechanism occurring when task demands increase (e.g., when the execution of a transitive action is contrasted with its mere observation), and information processing within distributed functional neural networks have to be enhanced. In this vein, it has been previously proposed that the neural synchronization at delta and theta frequencies (<8 Hz) might subserve a long-range coordination of distant brain regions, while neural synchronization at higher frequencies would subserve local interactions of neural assemblies (Kopell et al., 2000; von Stein and Sarnthein, 2000; Siegel et al., 2012). Furthermore, it has been proposed that cortical theta rhythms reveal integration of motor commands and incoming sensory information for an on-line correction of the motor output (Bland and Oddie, 2001; Caplan et al., 2003; Ekstrom et al., 2005), the so-called sensorimotor integration hypothesis.

The sensorimotor integration hypothesis is supported by several lines of evidence. Previous ECoG recordings in rodents have shown that during motor acts, theta rhythms are often coherent between the hippocampus and prefrontal cortex (Jones and Wilson, 2005) and between the hippocampus and parietal cortex (Sirota et al., 2008). Furthermore, a recent scalp EEG study in humans has reported increased theta rhythms accompanying movement initiation and sensory processing without any motor learning or memory demand (Cruikshank et al., 2012). Moreover, such modulation of theta rhythms was distinct from purely motor-related desynchronization of mu rhythm (Cruikshank et al., 2012). As scalp EEG primarily reflects neocortical sources activated from cortical and sub-cortical signals (e.g., brainstem, thalamus, basal ganglia and hippocampus; Nunez and Silberstein, 2000) and frontoparietal lesions impair sensorimotor information processing (Sirigu et al., 2004), it has been hypothesized that sensorimotor integration is an emerging cortical function (Ekstrom and Watrous, 2014). Sensorimotor-related EEG oscillations may manifest in the hippocampus via phase synchronization with cortical and subcortical brain areas (Ekstrom and Watrous, 2014).

Taking the above theoretical framework, the present findings represent the first demonstration of a neurophysiological mechanism exploiting delta-theta synchronization for enhancing the functional connectivity between lateral premotor and ventral prefrontal areas during the execution of reaching and grasping movements with respect to the mere observation of the same actions performed by someone else. Another preliminary result of the present study indicated that the phase of the delta/theta rhythms in the lateral premotor area would entrain the amplitude of the alpha, beta and gamma rhythms in the ventral prefrontal cortex during the action execution, but not during its observation. This finding complements and extends previous ECoG evidence in epilepsy patients showing that the alpha or beta phases entrained the amplitude of gamma rhythms in the very early stages of movement preparation (Miller et al., 2012; Yanagisawa et al., 2012).

## Conclusion

In the present study, ErLLC of ECoG rhythms was estimated in a small group of drug-resistant epilepsy patients in order to assess the hypothesis that the functional relationships between sensorimotor cortical regions would be enhanced during reaching and grasping of objects in the first person compared to the mere observation of those actions performed by someone else. We found that the ErLLC of delta-theta rhythms between the ventral prefrontal and lateral premotor areas was greater during action execution than its observation. Furthermore, the phase of those rhythms entrained the amplitude of the coupling of ECoG rhythms with higher frequencies such as alpha, beta and gamma.

These results support the validity of our experimental approach based on pre-surgical ECoG recordings from epilepsy patients, and the computation of ErLLC of ECoG rhythms for probing functional cortical connectivity underpinning the execution and the observation of reaching and grasping movements in humans. All in all, we suggest that during the execution of transitive actions, neurophysiological low-frequency oscillatory mechanisms operating in a frontal neural network formed by BA6 and BA44/45 would underpin the integration of motor commands and visual-somatosensory feedback accompanying the own performance. Future studies will have to confirm this proof-of-concept exploiting a large sample of epilepsy patients and more conservative statistical models.

## Ethics Statement

This study was carried out in accordance with the recommendations of IRCCS Neuromed committee with written informed consent from all subjects. All subjects gave written informed consent in accordance with the Declaration of Helsinki. The protocol was approved by the IRCCS Neuromed committee. This study was approved by the IRCCS Neuromed, Pozzilli, Italy.

## Author Contributions

CB: project idea and writing article; CDP and SL: data analyses, estimation of functional connectivity of ECoG rhythms and writing article; GN and AS: data analyses, estimation of functional connectivity of ECoG rhythms; LP: localized electrode positions; GG, PPQ, RM and VE: patients selection and handling; VG: project idea; GM: project idea, EEG data recording and writing article.

## Conflict of Interest Statement

The authors declare that the research was conducted in the absence of any commercial or financial relationships that could be construed as a potential conflict of interest.
